# Influence of tooth position on wind instrumentalists’ performance and embouchure comfort: A systematic review

**DOI:** 10.1007/s00056-018-0128-2

**Published:** 2018-03-12

**Authors:** F. N. van der Weijden, R. B. Kuitert, F. R. U. Berkhout, G. A. van der Weijden

**Affiliations:** 1 Academic Centre for Dentistry Amsterdam (ACTA), University of Amsterdam and VU University Amsterdam, Gustav Mahlerlaan 3004, 1081 LA Amsterdam, The Netherlands; 2 Department of Orthodontics, Academic Centre for Dentistry Amsterdam (ACTA), University of Amsterdam and VU University Amsterdam, Gustav Mahlerlaan 3004, 1081 LA Amsterdam, The Netherlands; 3 Implantology Amsterdam, Stadionweg 35-hs, 1077 RW Amsterdam, The Netherlands; 4 Department of Periodontology, Academic Centre for Dentistry Amsterdam (ACTA), University of Amsterdam and VU University Amsterdam, Gustav Mahlerlaan 3004, 1081 LA Amsterdam, The Netherlands

**Keywords:** Dentition, Overbite, Music, Malocclusion, Dental arch, Gebissform, Überbiss, Musik, Malokklusion, Zahnbogen

## Abstract

**Purpose:**

To systematically search the scientific literature concerning the influence of tooth position on wind instrumentalists’ performance and embouchure comfort.

**Methods:**

The PubMed, Cochrane, and Embase databases were searched up to November 2017. The main orthodontic journals were searched for papers older than the inception date of PubMed. Grey literature was sought via Google Scholar. Eligible studies were critically appraised and analysed.

**Results:**

The searches retrieved 54 papers. Only two met the inclusion criteria. Searching the orthodontic journals and Google Scholar resulted in two additional eligible studies. All four studies had a cross-sectional design. The sample sizes ranged from 20–100 participants, varying from children to professional musicians. Because of a large heterogeneity in outcome variables, no meta-analysis could be performed. Descriptive analysis shows that there are indications that tooth irregularities have a negative influence on embouchure comfort and performance of a wind instrument player. A large overjet may impede the embouchure of brass musicians and may have a negative influence on trumpet player performance. A wide jaw form seems more beneficial to trumpet player performance than a small jaw form. Furthermore, players of all types of wind instruments can experience embouchure difficulties from extreme spacing or an open bite.

**Conclusion:**

Tooth position can influence musical performance and embouchure comfort of wind instrumentalists. A Class I relationship without malocclusion seems appropriate for every type of wind instrument. The more extreme the malocclusion, the greater the interference with wind instrumentalists’ performance and embouchure comfort. Evidence however is limited.

**Electronic supplementary material:**

The online version of this article (10.1007/s00056-018-0128-2) contains supplementary material, which is available to authorized users.

## Zusammenfassung

**Ziel:**

Ziel der vorliegenden Studie war die systematische Suche nach wissenschaftlicher Literatur zum Einfluss der Zahnstellung auf die Leistung von Blasinstrumentalisten und den Komfort beim Ansatz des Instruments.

**Methoden:**

Die Datenbanken PubMed, Cochrane und Embase wurden bis 11/2017 durchsucht. Darüber hinaus wurden die wichtigsten kieferorthopädischen Zeitschriften nach Artikeln durchsucht, die vor der Einführung von PubMed publiziert worden waren. Die graue Literatur wurde über Google Scholar durchsucht. Thematisch passende Studien wurden kritisch beurteilt und analysiert.

**Ergebnisse:**

Die Suche führte zu 54 Veröffentlichungen, von denen nur 2 die Einschlusskriterien erfüllten. Durch weitere Suche in kieferorthopädischen Zeitschriften und Google Scholar wurden jeweils 2 weitere passende Studien gefunden, alle 4 waren Querschnittsstudien. Die Stichprobengröße reichte von 20–100 Teilnehmenden und von Kindern bis zu professionellen Musikern. Aufgrund einer großen Heterogenität der Ergebnisvariablen konnte keine Metaanalyse durchgeführt werden. Die deskriptive Analyse ergibt Hinweise darauf, dass eine unregelmäßige Zahnstellung einen negativen Einfluss auf den Komfort des Ansatzes der Instrumente und auf die Leistung von Blasinstrumentalisten hat. Eine vergrößerte sagittale Frontzahnstufe kann den Ansatz von Blechblasinstrumenten erschweren und die Leistung von Trompetenspielern beeinträchtigen. Eine breite Kieferform scheint für Trompetenspieler vorteilhafter zu sein als eine schmale Kieferform. Darüber hinaus führen bei Spielern aller Arten von Blasinstrumenten große Lücken und ein offener Biss zu Schwierigkeiten beim Ansatz.

**Schlussfolgerung:**

Die Zahnstellung kann bei Blasinstrumentalisten die musikalische Leistung und den Komfort beim Ansatz beeinflussen. Eine Klasse-I-Okklusion scheint für jede Art von Blasinstrument vorteilhaft. Je extremer die Malokklusion ist, desto mehr wird die Leistung und der Komfort des Ansatzes bei Blasinstrumentalisten beeinträchtigt. Die Evidenz ist jedoch begrenzt.

## Introduction

For musicians of wind instruments, the teeth contribute to the sound produced by their instrument. Teeth give support to the lips, cheeks and tongue, and therefore partly determine the tone production. The whole complex of anatomical structures around the mouth and the way they are used for playing the wind instrument is called the “embouchure”. The three major components of embouchure are the tongue, the teeth and the muscles of cheek and lip. Aspects like timbre (e.g., round or sharp tone), but also volume, intonation, phrasing and articulation (staccato, legato) are obviously determined by the quality of the instrument and mouthpiece or reed, but even more by the embouchure [2]. Different mouthpieces on wind instruments each require a specific technique to form the embouchure [22]. However, individuals will develop their own unique habitual muscular pattern which will vary in small detail between each player [17]. Since the personalized embouchure varies between musicians the sound of each player will—even on the same instrument—be quite different [2].

For optimum respiratory comfort, all wind musicians instinctively choose a mouthpiece position on or between the lips (depending on the instrument) where the passage of the air column is easiest. At the same time the player chooses a position for the mouthpiece where, from experience, maximal lip and dental comfort is achieved. This position becomes habitual from early training and embouchure development after several years of study and practice [16]. Embouchure comfort is necessary for efficient performance. Embouchure discomfort will disturb the player unduly and may affect the “tone” and limit the scope for artistic interpretation [16].

Because orthodontists are likely to be in contact with young wind musicians, Howard et al. [7] approached 160 orthodontists in Washington state with the request to fill out a questionnaire. The results show that only 23% inquire whether new patients are currently playing or are considering playing a wind instrument. The orthodontists estimated that 7% of their patients are wind musicians. An understanding of the relationship between wind instrument mouthpieces and dental and skeletal structures was deemed inadequate by 78% of the respondents. When questioned specifically about the relationship of each type of mouthpiece to different malocclusions, 34% of the respondents stated that they were not sure how to advise a patient. Approximately one quarter of the orthodontists could remember a patient ever having sought orthodontic therapy because they thought it might improve their comfort or ability when playing a wind instrument.

The majority of the literature concerning this topic is comprised of narrative reviews. The aim of this systematic review was to comprehensively search the scientific literature, identify, appraise and synthesize studies concerning the influence of an abnormal tooth position in comparison to a normal tooth position on musical performance and embouchure comfort in wind instrumentalists.

## Methods

### Protocol

The recommendations for strengthening the reporting were followed in accordance with the Preferred Reporting Items for Systematic Reviews and Meta-Analyses (PRISMA) statement [1]. The protocol of this systematic review detailing the review method was developed *a priori* following initial discussions between members of the research team.

### Focused questions (PICOS) and eligibility criteria

In observational studies and (randomized) controlled clinical trials, what is the difference in performance (primary outcome) and embouchure comfort (secondary outcome) between wind musicians with a normal tooth position and those with an abnormal tooth position?

The following criteria were imposed for inclusion in the systematic review:

- All studies describing the effect of tooth position on wind instrumentalists’ performance or embouchure comfort.

The exclusion criteria were as follows:

- Editorial letters, narrative reviews and case reports.
- Studies regarding non-wind musicians or singers.
- Studies regarding the influence of playing a wind instrument on the tooth position.

### Information sources and search

The PubMed, Cochrane and Embase databases were searched from initiation up to November 2017 (Table 1; F.N.W. and D.E.S.). Furthermore, the main orthodontic journals (*European Journal of Orthodontics* [Volume 1, Issue 1, January 1979–Volume 39, Issue 5, October 2017]; *American Journal of Orthodontics and Dentofacial Orthopedics* [Volume 1, Issue 1, January 1915–Volume 152, Issue 5, November 2017]; *Angle Orthodontist* [Volume 1, Issue 1, January 1931–Volume 87, Issue 6, November 2017]) were searched for papers older than the inception date of PubMed using the search engine as provided by these journals (R.B.K and F.N.W.). Also grey literature was sought via Google Scholar (using various combinations of the following keywords: tooth position, malocclusion, wind instrument, musical instrument, performance). In addition, the reference lists of all selected studies were hand searched for additional relevant articles (F.N.W. and G.A.W.).

**Table 1:** Keywords and search strategy

$$(<"\text{Malocclusion}"[\text{MESH}]\text{ OR malocclusion}> \text{OR} <"\text{Dental Occlusion}"[\text{Mesh}] \text{OR occlusion}> \text{OR} <"\text{Orthodontics}" [\text{MeSH}] \text{OR orthodontic}*>\text{ OR} <\text{tooth AND position}*>)$$
AND
$$ (<\text{instrument AND} \{"\text{Music}"[\text{MeSH}] \text{OR Music}\}> \text{OR} <\text{wind AND instrument}* \text{AND music}>) $$

The asterisk was used as a truncation symbol

### Study selection

Titles and abstracts of the studies obtained from the searches were screened independently by two reviewers (F.N.W. and G.A.W.) and were categorized as definitely eligible, definitely not eligible, or questionable. No language restrictions were imposed. No attempt was made to blind the reviewers to the names of authors, institutions, or journals while making the assessment. If eligible aspects were present in the title, the paper was selected for further reading. If none of the eligible aspects were mentioned in the title, the abstract was read in detail to screen for suitability. Papers that could potentially meet the inclusion criteria were obtained and read in detail by the two reviewers (F.N.W. and G.A.W.). Disagreements in the screening and selection process concerning eligibility were resolved by consensus or, if disagreement persisted, by arbitration through a third reviewer (R.B.K.). The papers that fulfilled all of the inclusion criteria were processed for data extraction.

### Data collection process, summary measures and synthesis of results

When provided, information about the characteristics of the study sample population, assessed parameters, conditions and outcomes were extracted from all the studies by two authors (F.N.W. and G.A.W.). As a summary, first a descriptive data presentation was used for all studies. In order to provide an overview of features of malocclusion connected to specific problems regarding playing different wind instruments, the outcomes of the selected studies were categorized by the same authors. Categorization was confirmed with a third author (R.B.K.). The outcomes were collected in a table sorted by the condition (jaw relationship, jaw form and tooth position). If possible, the data from the included studies were synthesized into a meta-analysis. As planned *a priori*, relative to the type of wind instrument, a subgroup analyses was conducted.

### Risk of bias in individual studies

Two reviewers (F.N.W. and G.A.W.) scored the individual methodological qualities and potential risk of bias of the included studies using a comprehensive combination of the Critical Appraisal Checklist for Analytical Cross Sectional Studies, developed by the Joanna Briggs Institute [11], the Newcastle Ottawa scale adapted for cross-sectional studies [8] and the ROBINS-I tool [4].

### Risk of bias across studies

Factors used to evaluate the clinical heterogeneity of the characteristics of the different studies were as follows: study design, participants, variables used to measure performance and embouchure comfort, variables used to assess tooth position.

### Rating the certainty of the evidence (GRADE)

The Grading of Recommendations Assessment, Development and Evaluation (GRADE) system was used, as proposed by the GRADE working group, to appraise the evidence emerging from this review. Two reviewers (F.N.W. and G.A.W) rated the strength of the evidence according to the following aspects: risk of bias, consistency of results, directness of evidence, precision, publication bias and magnitude of the effect [9, 10]. Any disagreement between the two reviewers was resolved after additional discussion.

## Results

### Study selection

**Fig. 1:**
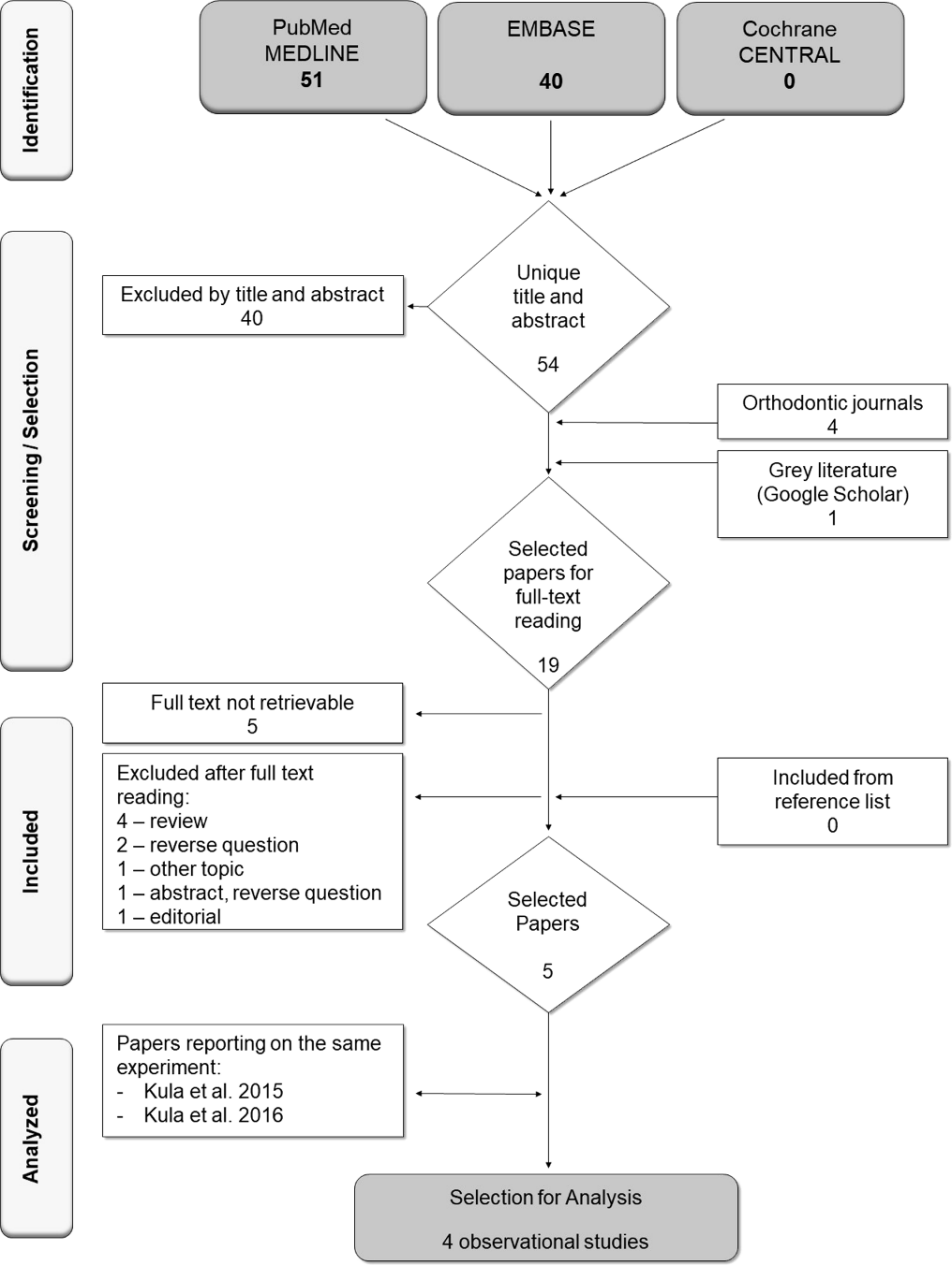
Flowchart of search and selection

The searches in PubMed, Cochrane and Embase resulted in 54 unique papers (Fig. 1). The screening of the titles and abstracts resulted in 14 potentially eligible papers. The full text of five papers was not retrievable. For nine papers the full texts were obtained and read in full. Of these, three [12, 15] met the eligibility criteria. However, two of these papers reported on the same experiment. Searching the main orthodontic journals revealed four potentially eligible papers of which one was selected [3]. Google scholar also yielded one suitable paper [13]. Screening the reference lists of the four selected full-text papers resulted in no additional paper. Subsequently, a total of four papers (Table 2) were included in this systematic review.

**Table 2:** Study characteristics of included papers

Authors	Year of publication	Geographic location	Study design	Sample size	Population	Age	Male/Female
Lamp and Epley [13]	1935	USA	Cross-sectional	62	14 or 15 year old children	14–15 years old	Not applicable
Cheney [3]	1947	USA	Cross-sectional	100	Members of university bands, students at the conservatory and music teachers	Not applicable	Not applicable
Lovius and Huggins [15]	1973	UK	Cross-sectional	20	Professional orchestra musicians	31.8 ± 7.1 years old	15 male, 5 female
Kula et al. [12]	2016	USA	Cross-sectional	70	University students	22.2 ± 3.8 years old	Not applicable

### Study characteristics

All four eligible papers had a cross-sectional study design. Three originated from the USA and one from the UK. The sample sizes ranged from 20–100 participants. The population characteristics varied from children to professional musicians.

### Risk of bias within studies

To estimate the potential risk of bias, the methodological qualities of the included studies were assessed (Table 3). Lip form, lip thickness and lip closure as potential confounding factors were defined and assessed in three of the four studies [3, 13, 15], but were analysed in only one [13]. In three of the four studies [3, 12, 15] the sample was clearly defined and representative. Measurement of the tooth position was valid and reliable in three of the four studies [3, 12, 15]. In two of the four studies [3, 12] assessment of performance or embouchure comfort was measured in a valid and reliable way. Overall, the potential risk of bias of the included studies was estimated to be “serious” for one study [13] and “moderate” for the other three [3, 12, 15]. Because only four papers were identified, which provided heterogeneous outcome parameters, reporting bias could not be assessed nor was a sensitivity analysis feasible.

**Table 3:** Risk of bias assessment using a comprehensive combination of criteria as suggested by the critical appraisal checklist for analytical cross-sectional studies (Joana Briggs Institute; [11]), the Newcastle Ottawa scale adapted for cross-sectional studies [8] and the ROBINS-I tool (Risk Of Bias In Non-randomized Studies—of Interventions as provided by Cochrane; [4]). Judgement of risk of bias is presented according to the 7 domains as suggested by the ROBINS-I tool

	Lamp and Epley [13]	Cheney [3]	Lovius and Huggins [15]	Kula et al. [12]
**Pre-assessment domains**				
1. Bias due to confounding				
Were confounding factors defined?	Yes	Yes	Yes	No
Were confounding factors assessed?	Yes	Yes	Yes	No
Were strategies to deal with confounding factors stated?	Yes	No	No	No
*Risk of bias judgement*	*Low*	*Moderate*	*Moderate*	*Critical*
2. Bias in selection of participants into the study				
Were the criteria for inclusion in the sample clearly defined?	No	Yes	Yes	Yes
Were the study subjects described in detail?	No	Yes	Yes	Yes
Is the study sample representative of the average in the target population?	No	Yes	Yes	Yes
Is the sample size justified and satisfactory?	No	No	No	No
*Risk of bias judgement*	*Critical*	*Moderate*	*Moderate*	*Moderate*
3. Bias in classification of condition (tooth position)				
Were objective, standard criteria used for measurement of the condition?	Yes	Yes	Yes	Yes
Is the measurement tool validated?	No	Yes?^1^	Yes	Yes
Was the condition assessed in a reliable way?	No	Yes	Yes	Yes
*Risk of bias judgement*	*Serious*	*Low*	*Low*	*Low*
**Post-assessment domains**				
4. Bias due to deviations from intended intervention				
*Risk of bias judgement*	N/A	N/A	N/A	N/A
5. Bias due to missing data				
*Risk of bias judgement*	N/A	N/A	N/A	N/A
6. Bias in measurement of outcomes				
Were the investigators blinded to the condition?	N/I	N/A	N/A	N/I
Were the outcomes measured in a valid and reliable way?	N/I	Yes	N/A	Yes
Was appropriate statistical analysis used?	Yes	No	No	Yes
*Risk of bias judgement*	*Serious*	*Serious*	*Critical*	*Moderate*
7. Bias in selection of the reported result				
Are the reported effect estimates based on the results?	Yes	Yes	Yes	Yes
*Risk of bias judgement*	*Low*	*Low*	*Low*	*Low*
**Overall risk of bias**	Serious	Moderate	Moderate	Moderate

*Judgements:* Low, moderate, serious, critical, no information (N/I), not applicable (N/A)

^1^Systematic oral examination record form used in the Orthodontic Clinic at the University of Michigan

### Results of individual studies

#### Study design and study authors’ conclusions

In the study of Lamp and Epley [13], 62 children (14–15 years old) were tested for their aptitude for brass and woodwind musical instruments after a controlled try-out period to these instrument types. A tooth evenness scale was constructed by taking three pictures of the anterior teeth (frontal opened, frontal closed and in profile) of the subjects by an expert photographer. The results showed that there is no relationship between tooth evenness and successful performance on brass or woodwind musical instruments.

In the study of Cheney [3] 100 wind musicians (36 small brass, 26 large brass and 38 woodwind musicians) were selected from members of the University of Michigan bands, students in the University School of Music, and music teachers in Ann Arbor in 1943–1944. Features of malocclusion and lip form were recorded and embouchure discomfort were evaluated via a questionnaire with open questions. The authors concluded that it is evident that there are many dentofacial irregularities which interfere with wind instrument musicianship.

Lovius and Huggins [15] examined 20 musicians (15 male, 5 female, 31.8 ± 7.1 years old) of the Royal Liverpool Philharmonic Orchestra playing various wind instruments. Each player was examined clinically and plaster models were obtained. The authors concluded that a sound dentition with minimal malocclusion is of such importance that without it a professional wind instrumentalist is unlikely to reach a sufficiently high standard to play in the leading orchestras of the UK.

In the study of Kula et al. [12] 70 trumpet students (22.2 ± 3.8 years old) of 11 universities were asked to play a scripted performance skills test of flexibility, articulation, range and endurance exercises using their own instrument. An experienced music teacher-investigator evaluated all performances. A three-dimensional cone-beam computed tomography (3D CBCT) scan was taken of each student the same day as the skills test. From this scan the following parameters were measured: occlusion, overjet, overbite, interincisor inclination, molar and canine inclination, degree of anterior tooth irregularity—Little’s irregularity index [14], rotation of the maxillary centrals, diastema, maxillary and mandibular intermolar and intercanine widths. The results of this study show that orthodontic problems may impede trumpet playing performance of young musicians. However, not all parameters of malocclusion were found to be associated with trumpet player performance.

#### Study outcomes

Data extracted from the included studies are presented in Tables 4, 5 and 6. These tables provide details about features of malocclusion related to specific problems on playing various wind instruments concerning performance and embouchure. Because the four studies show a large heterogeneity in outcome variables no meta-analysis could be performed. Therefore, a descriptive method was chosen to summarize and analyse the data.

**Table 4:** Extracted data on primary and secondary outcome related to jaw relationship and type of wind instrument. Studies that support the observations are indicated

	Type of wind instrument	Influence on performance or embouchure comfort	Study
Molar classification and overjet	Trumpet	No effect on performance	Kula et al. [12]
	All	All players with normal occlusion do not experience embouchure difficulties	Cheney [3]
	All	Class III (reversed overjet) malocclusion embouchure difficulties are distributed evenly among woodwind, small^1^ and large^2^ brass musicians	Cheney [3]
	Brass	Six musicians complained of inability to shift the lower jaw forward. Five were (mostly small^1^) brass musicians, with Class II malocclusion (enlarged overjet). Class II arch relationships of one cusp or more (large overjet) appear more troublesome than discrepancies of one-half cusp or less (mild overjet). Six musicians complained of unsatisfactory adjustment to embouchure, but were unable to identify the cause of poor adjustment. They all had extreme disto-occlusions, except for one that had Class II arch relationship of one-half cusp but mildly protruding maxillary incisors and a short upper lip	Cheney [3]
	Small brass	Among players with a Class I malocclusion, only small brass players experience embouchure difficulties	Cheney [3]
	Woodwind	Disto-occlusion does not interfere with embouchure	Cheney [3]
Overbite	Trumpet	No effect on performance	Kula et al. [12]
	All	In Class II: The deeper the overbite, the greater the tendency for embouchure difficulties	Cheney [3]
Open-bite	Brass	Extreme open bite seriously interferes with embouchure	Cheney [3]
	Woodwind	Little effect on embouchure comfort, except for a partial anterior open-bite opposite the corner of the mouth (infraocclusion of maxillary canines and lateral incisors). These musicians experience difficulty in preventing the escape of air through the corners of the mouth	Cheney [3]
Crossbite of anterior teeth	All	With full crossbite (all upper incisors lingual to lower incisors) no embouchure difficulties	Cheney [3]
	Brass	With a single crossed incisor adjustment of the small^1^ brass mouthpiece against the lip was often difficult. Brass musicians with this irregularity complain that it forces them to replace the instrumental mouthpiece unevenly against the lip	Cheney [3]
	Woodwind	Woodwind musicians with this irregularity complain that it irritates the lower lip	Cheney [3]

^1^ Small brass = trumpet, bugle, French horn and alto horn

^2^ Large brass = trombone, baritone, bass horn, tuba

**Table 5:** Extracted data on primary and secondary outcome related to tooth position and type of wind instrument. Studies that support the observations are indicated

	Type of wind instrument	Influence on performance or embouchure comfort	Study
Anterior tooth irregularity	Brass and woodwind	No relationship between tooth evenness and successful performance	Lamp and Epley [13]
	All	All musicians with extreme anterior crowding experienced embouchure difficulties. They complained that the sharp corners of the rotated crowns irritated the lips	Cheney [3]
Anterior maxillary tooth irregularity	Trumpet	Significant negative relation with double tongue^b^ articulation	Kula et al. [12]
	All	Fourteen of the 36 musicians with upper crowding experienced embouchure difficulties; only five complained of the irregularity as the direct cause	Cheney [3]
	Brass	In combination with disto-occlusion more than half of the brass musicians experienced embouchure difficulties	Cheney [3]
	Brass	In combination with Class III malocclusion all brass musicians experience embouchure difficulties	Cheney [3]
Interincisal rotation of the maxillary centrals	Trumpet	Significant negative relation with flexibility^a^ exercise	Kula et al. [12]
Anterior mandibular tooth irregularity	Trumpet	Significant negative relation with flexibility^a^ exercise and double tongue^b^ articulation	Kula et al. [12]
	All	Embouchure difficulties more often in combination with mesio- or disto-occlusion 12 of the 40 individuals with mandibular crowding experienced embouchure difficulties; only 3 of them complained of the irregularity as the direct cause	Cheney [3]
	Brass	Occasionally embouchure difficulties	Cheney [3]
	Woodwind	Often troublesome	Cheney [3]
Protrusion of upper incisors	Trumpet	Significant negative relation with flutter tongue^b^ articulation	Kula et al. [12]
	Brass	Embouchure difficulties with maxillary protrusion	Cheney [3]
	Woodwind	No embouchure difficulties with maxillary protrusion	Cheney [3]
Retrusion of upper incisors	All	Retrusion of all upper incisors (without crowding) did in no case present embouchure difficulties	Cheney [3]
	Brass	In combination with Class II relationship retrusion of the upper incisors is of advantage for the brass player	Cheney [3]
Retrusion of lower incisors	Woodwind	Embouchure difficulties with retrusion of lower incisors	Cheney [3]
Mid diastema	Trumpet	No effect on performance	Kula et al. [12]
Anterior spacing	All	All musicians with extreme anterior spacing experienced embouchure difficulties. For all these individuals, the problems centred around discomfort and pain of the teeth and supporting bone and/or early fatigue and pain of the muscles of the floor of the mouth and lip	Cheney [3]

^a^Flexibility was evaluated using three exercises: moving up/down between adjacent partials, slurring nonadjacent partials up/down and alternating between adjacent intervals. The speed/tempo was measured using a metronome program on a laptop computer

^b^Articulation evaluated different tongue movements using four exercises: single tongue (producing the sound “ta”), double tongue (“ta ka”), triple tongue (“ta da ka”) and flutter tongue (“trrr”)

**Table 6:** Extracted data on primary outcome related to jaw form and type of wind instrument. Studies that support the observations are indicated

	Type of wind instrument	Influence on performance	Study
Maxillary intercanine width	Trumpet	Significant positive relation with flutter tongue^b^ articulation	Kula et al. [12]
Maxillary intermolar width	Trumpet	Significant positive relation with flutter tongue^b^ articulation	Kula et al. [12]
Mandibular intercanine width	Trumpet	No effect on performance	Kula et al. [12]
Mandibular intermolar width	Trumpet	Significant positive relation with flexibility^a^ exercise, triple and flutter tongue^b^ articulation	Kula et al. [12]

^a^Flexibility was evaluated using three exercises: moving up/down between adjacent partials, slurring nonadjacent partials up/down and alternating between adjacent intervals. The speed/tempo was measured using a metronome program on a laptop computer

^b^Articulation evaluated different tongue movements using four exercises: single tongue (producing the sound “ta”), double tongue (“ta ka”), triple tongue (“ta da ka”) and flutter tongue (“trrr”)

### Synthesis of results

#### Performance

Lovius and Huggins [15] found that out of 20 professional orchestra wind instrument players 50% had Class I, 25% had Class II/1, 5% Class II/2 and 20% Class III relationship. As only half of the participants had a Class I relationship, a neutro-occlusion does not seem to be a prerequisite in order to reach a professional level of performance on the wind instrument. However, the authors [15] noted that the subjects that deviated from a Class I occlusion had minimal malocclusions. For instance, Class III subjects showed reduced overbite and overjet or edge-to-edge incisor relationship, rather than a reversed overjet.

The study of Kula et al. [12] does not report a negative effect of a Class II or III relationship on trumpet player performance (Table 4). However, maxillary and mandibular molar arch width and maxillary canine arch width were positively related to trumpet player performance (Table 6). On the other hand, maxillary protrusion and anterior crowding are found to have a negative effect (Table 5). The latter is not in agreement with Lamp and Epley [13] who found no relationship between tooth evenness and successful performance on brass or woodwind musical instruments.

#### Embouchure comfort

Cheney [3] found that none of the players with a normal occlusion (Class I without malocclusion) experiences embouchure difficulties. Embouchure difficulties occur most frequently among musicians with Class II malocclusion who play a small brass instrument (Table 4). Difficulties appear relative in proportion to the extent of overjet (disto-occlusion or protrusion of maxillary incisors; [3]; Tables 4 and 5). Furthermore, Cheney [3] found a negative effect of anterior crowding on embouchure comfort. Most often problems occur when the crowding is extreme or in combination with disto- or mesio-occlusion. Opposite to crowding, all musicians with extreme anterior spacing also experienced embouchure difficulties ([3]; Table 5). For brass musicians, an extreme open bite seriously interferes with embouchure, whereas woodwind instrumentalists experience little negative effect of an open bite, except for a partial anterior open-bite opposite the corner of the mouth ([3]; Table 4).

### Rating the certainty of the evidence (GRADE)

Table 7 presents a summary of the various factors used to rate strength of the evidence according to GRADE [9, 10]. Although most studies examined advanced or professional wind instrumentalists, the generalizability is limited because most studies have looked at woodwind musicians as one group. Although the outcomes of the included studies are rather consistent, the measurement of these outcomes was inexact. Furthermore, the potential risk of bias is estimated to be moderate to serious. Reporting bias cannot be assessed but can also not be ruled out. In the absence of distinct end points the magnitude of the effect is undeterminable. Therefore, the strength of the evidence emerging from this systematic review was estimated to be “very weak”.

**Table 7:** Summary of the estimated evidence profile (GRADE; [9, 10])

Determinants of quality	Overall
Study design	Observational
Number of studies	4
Risk of bias	Moderate to serious
Consistency	Rather consistent
Directness	Limited generalizability
Precision	Inexact
Reporting bias	Cannot be ruled out
Magnitude of the effect	Undeterminable
Strength of the evidence	Very weak

## Discussion

### Answer to the focused question

This is the first systematic review that maps the influence of tooth position on wind instrumentalists’ performance and embouchure comfort. Little research appears to have been carried out in this field most of which is in the form of narrative reviews with the risk of being neither objective nor robust.

Most included studies regarded woodwinds as one group, although single reed-, double reed instrumentalists and flutists have a different embouchure (Table 8). In order to better comprehend the following paragraphs, it is useful to have knowledge of how different types of wind instruments are held in or against the mouth.

**Table 8:** How different types of wind instruments are held in or between or against the mouth ([20]; reprinted with permission from the *British Dental Journal*)

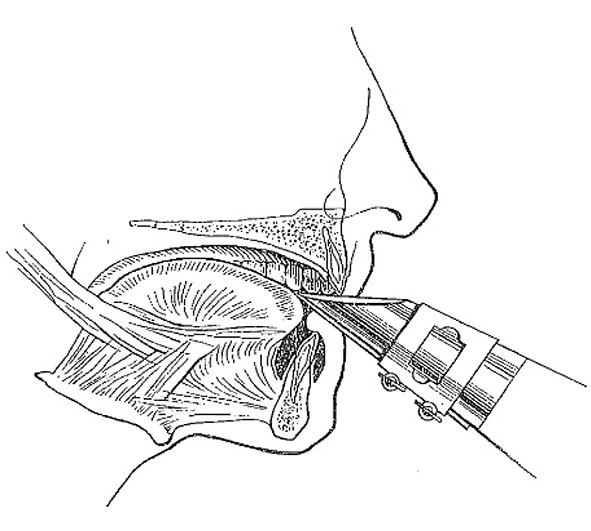	Single-reed instruments (clarinet, saxophone etc.) are played intra-orally with a wedge-shaped mouthpiece on which at the underside a reed is attached. The maxillary incisors rest on the sloping upper surface of the mouthpiece, while the lower lip is placed between the lower surface of the mouthpiece and the mandibular incisal edges (single-lip embouchure; [2, 22])
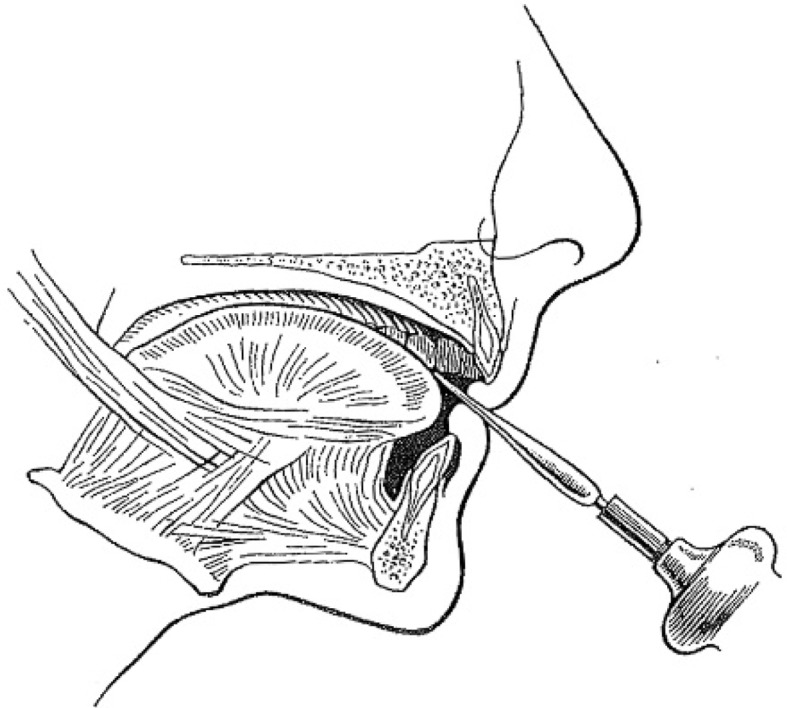	Double-reed instruments (oboe, bassoon etc.) are played intra-orally with a mouthpiece made from two bamboo reeds bound together with a cord. The reed is placed in the mouth, between the upper and lower lips, which covers the underlying incisal edges (double-lip embouchure; [2, 22])
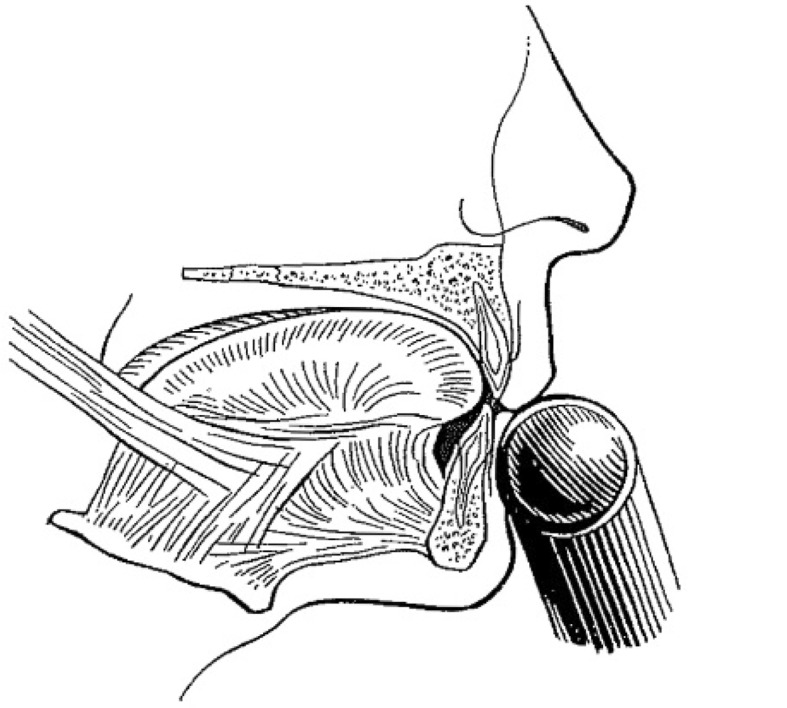	The flute or piccolo is played extra-orally by holding the mouthpiece against the lower lip, whereby the lower anterior teeth serve as a support. The upper lip is pushed downward to form a small slit-shaped opening between the lower and upper lip, which directs the air towards the opposite rim of the blowhole. The embouchure of the flute is partly controlled by the position of the flute in relation to the upper lip. This is done by a rotation movement of the flute in the plica mentalis in combination with protrusion and retrusion of the mandibula [2, 22]
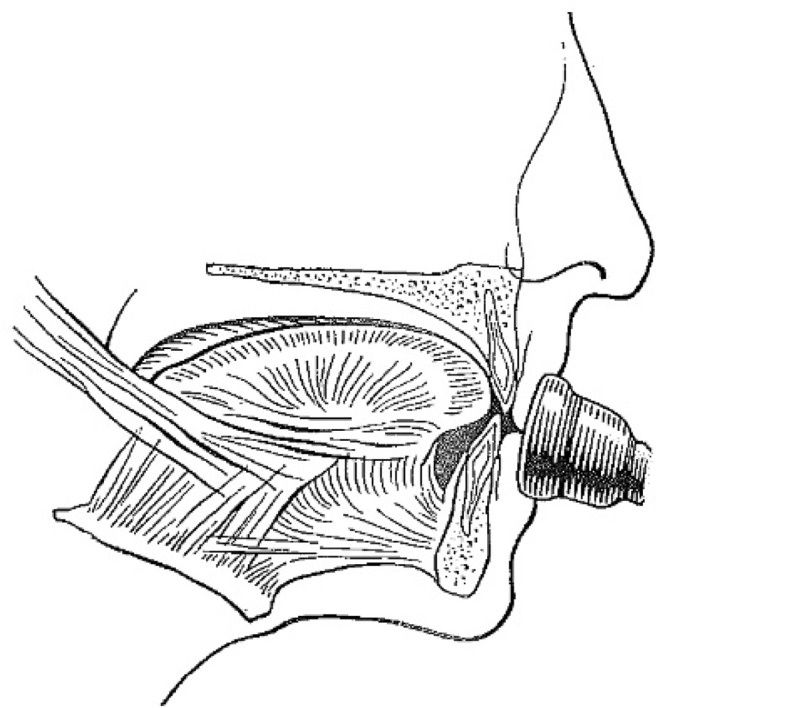	Brass instruments (trumpet, trombone, horn, tuba etc.) are played extra-orally by pushing the bowl-like mouthpiece against the upper and lower lip. Both upper and lower anterior teeth provide support for the lips. The lips are, depending on the height of the tone, pulled tight and set in vibration [2, 22]

A Class I relationship without malocclusion is appropriate for every type of wind instrument [3]. Mainly the extreme malocclusion results in embouchure difficulties [3]. Observation among professional orchestra members suggest that a Class I relationship does not seem to be a prerequisite in order to reach a professional level of performance on the wind instrument [15]. Furthermore, it might be concluded that as long as the malocclusion is mild it does not negatively influence wind instrumentalists’ performance to an extent that it interferes with a professional career.

Based on the outcomes of the included studies, limited evidence is available to provide a conclusive answer regarding positive or negative influences of specific tooth positions on the performance and embouchure comfort of musicians of different types of wind instruments.

### Angle class

In the literature it has been suggested that a Class I relationship is suitable to any type of wind instrument. For single-reed musicians, a Class II relationship supposedly is least troublesome [2, 5]. Berkhout [2] suggests that a Class II-1 is less ideal for brass and flute musicians and a Class II-2 is relatively unfavourable for most wind instruments. Because the instrument must be kept uncomfortably high with a Class III relationship this is suggested to be especially unfavourable for single reed instruments [2]. Also a protruding lower jaw is presumably a distinct disadvantage to brass instrument playing because the upper and lower lips should be in a straight vertical line with the mouthpiece applied to them perpendicularly [20].

From the evidence emerging of the selected studies, it indeed appears that a Class I relationship without malocclusion seems appropriate for every type of wind instrument [3]. According to the same study, extreme disto-occlusion (large overjet) clearly disturbs the embouchure of brass musicians, whereas a disto-occlusion does not interfere with woodwind embouchure [3]. Among individuals with Class III malocclusion (reversed overjet) embouchure difficulties are distributed evenly among woodwind, small and large brass musicians [3].

### Overbite and open bite

It has been suggested that a deep anterior overbite is troublesome for playing a single reed instrument [16, 22]. It is presumed that it would be easier to play a single reed instrument with an anterior open bite because of its large intraoral mouthpiece [5]. But an open bite can also prevent lip support in musicians of certain instruments [18].

From the selected studies it appears that an extreme open bite seriously interferes with the embouchure of brass musicians [3]. No effect of overbite on trumpet player performance was found [12]. But among musicians with a Class II malocclusion it appears that the larger the overbite, the greater the tendency for embouchure difficulties [3].

### Jaw form and tongue

In a narrative review the need of arch width between brass instruments and flute are compared [2]. The author suggests that for good tongue function and thus articulation and tone production on a brass instrument the internal width and shape of the upper dental arch is very important, whereas on the flute the contour and lingual width of the lower arch is more important.

One of the included studies [12] showed that trumpet players with a wider maxillary and mandibular molar arch and wider maxillary canine arch performed better on exercises such as triple tongue movement and flexibility.

### Anterior teeth irregularity

The literature suggests that for brass instrument players it is important that the vestibular surface of both lower and upper incisors are regular and flat because they support the lips and the mouthpiece. The same would apply to double-reed instruments because the incisal ridges of the teeth are covered by the lips and the mouthpiece rests between the lips. If the arch is irregular, large forces are exerted on the lips at the site of ectostematic teeth, which can lead to pain or irritation. For single reed instrument players the lower anterior teeth are of special importance because they support the instrument and determine the pressure on the reed. Irregularities and sharp edges can be very painful [2, 5, 6, 18–20, 22]. Flute playing may be disturbed by excessively irregular anterior teeth in the lower jaw [19, 22]. Also maxillary incisor irregularity is supposedly a handicap to flute playing [2].

One of the included studies [13] did not find evidence for a relationship between tooth evenness and successful performance on the brass and woodwind musical instruments. Two other authors [3, 12], however, did find a negative effect of anterior crowding on performance and embouchure comfort. Wind instrument players with anterior crowding complain that the sharp corners of the rotated crowns irritated the lips as they supported the instrumental mouthpiece [3].

### Protrusion and retrusion of anterior teeth

In the literature it has been suggested that protruded teeth can cause pain, like irregular teeth [5, 18, 22]. There is disagreement whether protrusion of maxillary incisors is disturbing to single-reed musicians [5, 22]. Whereas retrusion of mandibular incisors is presumably troublesome to single-reed musicians, retrusion of maxillary incisors would be troublesome to brass instrument playing [22].

From two of the included studies [3, 12] it appears that maxillary protrusion has a negative effect on trumpet player performance and may impede the embouchure of brass musicians. Retrusion of all upper incisors does not present embouchure difficulties. Rather, when a Class II arch relationship exists, it is likely of advantage to the brass player [3]. Woodwind musicians experience no embouchure difficulties with maxillary protrusion but do experience difficulties with retrusion of lower incisors [3].

### Diastemas

In the literature it has been suggested that diastemas between anterior teeth can be disturbing to double reed instrument players because the free corners of the incisors exposed by such space tend to irritate the lips. Where a space is substantially wider, such as between the upper central incisors, the upper lip could get stuck between the teeth during playing [5, 18, 19]. For brass musicians this can result in disturbance of lip vibration. Any eccentric position of the embouchure to avoid these problems may cause rapid tiring [2, 6, 22]. In single reed instruments diastemas between the lower anterior teeth can cause pain, whereas diastemas between the maxillary incisors result in “false air” and adversely affect the sound of flute playing [2].

One of the included studies [3] shows that all musicians with extreme anterior spacing experience embouchure difficulties. For all these individuals, the adaptation problems centred around discomfort and pain of the teeth and supporting bone and/or early fatigue and pain of the muscles of the floor of the mouth and lip. Another included study [12], however, did not find an effect of mid diastemas on trumpet player performance.

### Confounder: the role of the lips

Quantz [21] was the flute teacher of Frederick the Great of Prussia and as far as is known the first who formulated requirements that the dentition and lips had to meet in order to play well:

Straight teeth, which are neither too long nor too short; Not thick, but thin, smooth, and fine lips, which have neither too much nor too little flesh, and can close the mouth without compulsion.

The role of the upper lip is relatively more important in flute playing than with other wind instruments because it directs the airflow [2]. A short upper lip or long (or protruding) upper incisors are a great handicap to flute musicians [2, 5, 6]. In brass instrument playing lip length has been suggested as a possible cause of disturbance in lip vibration. With relatively short lips higher tones will be more difficult to produce than the lower tones because the higher tones require an air column of smaller diameter to pass through the lips than the lower tones [20]. In double reed instrument playing, the lips also play an important role because they should be long enough to be stretched over the incisal edges [2, 18, 19].

In this systematic review the role of the lips in playing wind instruments can be regarded as a confounding factor. Only one of the included studies [3] investigated lip length and lip thickness. Thick lips were observed in a majority of Class II brass musicians with embouchure difficulties. Thin lip form appeared among many Class III brass musicians with embouchure difficulties. In addition, a tendency for a short upper lip or longer lip form was noticed among the Class I brass musicians with embouchure difficulties. The author [3] concludes that the emerging evidence is not clear-cut and that it is difficult to estimate the role of lip size in adjustment to embouchure.

### Risk of bias

The potential risk of bias of the included studies is estimated to be moderate to serious. Except for Kula et al. [12], all studies have been written four to seven decades ago during which period the criteria for reporting were not as strict as in the current guidelines. This may be one of the reasons that has an impact on the estimated risk of bias. Whereas the study of Kula et al. [12] is quantitative with objective measurements, the study of Cheney [3] can be classified as qualitative. Across studies the evidence emerging from this systematic review is graded to be “very weak”.

### Limitations

A limitation of this systematic review is that only four eligible studies could be included. Although no language filter was used, the possibility exists that some non-English papers were not added to the databases used. Also four potentially interesting non-English paper titles were identified by the search, of which the abstract and full text were not retrievable.

All the included studies have their own limitations. While the objective and reliable assessment parameters of Kula et al. [12] are a strength, the validity of the measurements can be questioned. Flexibility, which was measured as the quickest tempo while playing three exercises, also depends on finger dexterity, articulation also depends on tongue speed and endurance also depends on lung capacity. This is supported by the fact that a few students could not perform all tests. The variable “adjustment to embouchure” used by Cheney [3] is valid, but the reliability can be questioned because it is based on the subjective experience of wind musicians. It is an original idea of Lamp and Epley [13] to examine children during their first contact with a wind instrument on their aptitude for brass or woodwind and determine if there might be an association with tooth irregularities. After a certain instruction and period of practice an aptitude test was taken, but the test criteria were not described. Lovius and Huggins [15] measured and described the prevalence of a number of dental variables among a group of professional wind musicians. However, they did not measure any outcome variable. They just assumed that musicians of Liverpool Philharmonic Orchestra had a high standard of musical performance. Although this assumption is reasonable, professional musicians can also experience difficulties with their embouchure.

### Generalizability

Most included studies have looked at woodwind musicians as one group. Kula et al. [12] only looked at trumpet musicians, Lamp and Epley [13] distinguished between brass and woodwind instruments, Cheney [3] further divided brass instruments into those with a small or large mouthpiece, and Lovius and Huggins [15] regarded all wind instruments as a single group. This is a shortcoming of all studies because brass, single reed, double reed instrumentalists and flutists have a different embouchure (Table 8).

### Future research

Instead of cross-sectional studies, studies comparing predefined groups with respect to wind instrumentalists’ performance or tooth position would contribute to a better understanding of the impact of tooth position on the tone production. Malocclusion, up to a certain extent, might be compensated with an adaptation of the peri-oral musculature. Therefore, it would be interesting to investigate the role of the lips and other facial muscles in wind instrumentalists’ performance. Perhaps electromyography can be used. In addition it would be interesting to know if wind instrument players can perform better or with less difficulty after orthodontic correction. Finally, considering the various forms of embouchure (Table 8), it is important for future research to investigate the various types of wind instruments separately.

Conversely it would be of interest to systematically synthesize the available literature on the influence of playing wind instrument on tooth position.

## Clinical relevance

### Scientific rationale for the study

Dental care professionals should be able to advise their patients who play a wind instrument about the effect of a correction of malocclusion on performance and comfort. Scientific evidence about the relationship of each type of instrumental mouthpiece to different malocclusions may aid in their recommendation.

### Principle findings

Based on the outcomes of the included studies no firm answer can be given as to what specific tooth positions have a positive or negative influence on the embouchure comfort and performance of musicians of different types of wind instruments. A Class I relationship without malocclusion seems appropriate for every type of wind instrument. When a wind instrumentalist has a mild malocclusion it does not appear to impede a professional career. The more extreme the extent of malocclusion, the greater the interference with a wind instrumentalists’ performance and embouchure comfort.

### Practical implications

(Semi-)professional wind instrument players may benefit from orthodontic therapy in case of a more extreme malocclusion. A young musician may experience problems wearing braces but the end result may help to improve the musical performance and embouchure comfort.

## Conclusion

Based on four observational studies with moderate to serious risk of bias, it is concluded that tooth position can influence musical performance and embouchure comfort of wind instrumentalists. The emerging evidence suggest that a Class I relationship without malocclusion seems appropriate for every type of wind instrument. Additionally, the more extreme the malocclusion, the greater the interference with the wind instrumentalists’ performance and embouchure comfort will be. The strength of evidence of these findings is graded to be “very weak”.

## Caption Electronic Supplementary Material

**Online supplement 1.** Full list of the citations of all studies identified from the literature search including their inclusion/exclusion status with reasons for exclusion.
