# Supplementary material for: Influence of tooth position on wind instrumentalists’ performance and embouchure comfort: A systematic review
Source: J Orofac Orthop. 2018 Mar 12;79(3):205–18. doi: 10.1007/s00056-018-0128-2 (PMC5954010; doi:10.1007/s00056-018-0128-2)
Supplement: Supplementary file 1 — Online supplement 1. Full list of the citations of all studies identified from the literature search including their inclusion/exclusion status with reasons for exclusion. [file 56_2018_128_MOESM1_ESM.docx]

**Online supplement 1.** Full list of the citations of all studies identified from the literature search including their inclusion/exclusion status with reasons for exclusion.

Identified by PubMed:

1. Amorim MI, Jorge AI (2016) Association between temporomandibular disorders and music performance anxiety in violinists. Occup Med (Lond). 66:558-563.

► Excluded based on title and abstract, because of addressing other topic

2. Shafi I, Welbury R (2015) Idiopathic Radiographic Apical Root Resorption in Wind Instrument Players. Dent Update. 42:972-976.

► Excluded based on title and abstract, because of addressing other topic

3. Kula K, Cilingir HZ, Eckert G, Dagg J, Ghoneima A (2016) The association of malocclusion and trumpet performance. Angle Orthod. 86:108-114.

► Included

4. Kula K, Cilingir HZ, Eckert G, Dagg J, Ghoneima A (2015) The association of malocclusion and trumpet performance. Angle Orthod. [Epub ahead of print]

► Excluded after full text reading, because this is the same paper as Kula et al. (2016).

5. Głowacka A, Matthews-Kozanecka M, Kawala M, Kawala B (2014) The impact of the long-term playing of musical instruments on the stomatognathic system - review. Adv Clin Exp Med. 23:143-146.

► Excluded based on title and abstract, because of being a review paper

6. Killion MC (2012) Factors influencing use of hearing protection by trumpet players. Trends Amplif. 16:173-178.

► Excluded based on title and abstract, because of addressing other topic

7. Grammatopoulos E, White AP, Dhopatkar A. (2012) Effects of playing a wind instrument on the occlusion. Am J Orthod Dentofacial Orthop. 141:138-145.

► Excluded based on title and abstract, because of addressing the reverse question

8. Rodríguez-Lozano FJ, Sáez-Yuguero MR, Bermejo-Fenoll A (2011) Orofacial problems in musicians: a review of the literature. Med Probl Perform Art. 26:150-156.

► Excluded based on title and abstract, because of being a review paper

9. Authors unknown (2009) Malocclusion and wind instruments investigated. Br Dent J. 207:414.

► Excluded after full text reading, because of being an editorial

10. Raney NA (2006) The effects of orthodontic appliances on wind-instrument players. J Clin Orthod. 40:384-387.

► Excluded based on title, because of addressing other topic

11. Raney N (2005) Impact of orthodontic braces on wind instrument players. Tex Dent J. 122:676-679.

► Excluded based on title, because of addressing other topic

12. Sbeity ZH, Mansour AM (2004) Recurrent retinal vein occlusion after playing a wind instrument. Graefes Arch Clin Exp Ophthalmol. 242:428-431.

► Excluded based on title and abstract, because of addressing other topic

13. Yeo DK, Pham TP, Baker J, Porters SA (2002) Specific orofacial problems experienced by musicians. Aust Dent J. 47:2-11.

► Excluded based on title and abstract, because of being a review paper

14. Green HM, Green SE (1999) The interrelationship of wind instrument technic, orthodontic treatment, and orofacial myology. Int J Orofacial Myology. 25:18-29.

► Excluded based on title and abstract, because of being a review paper

15. Feldmann H (1997) History of the tuning fork. II: Evolution of the classical experiments by Weber, Rinne and Schwabach. Laryngorhinootologie 76:318-326.

► Excluded based on title and abstract, because of addressing other topic

16. O'Brien M (1993) Orthodontic considerations in selecting wind instruments. J Gen Orthod. 4:11-13.

► Excluded because full text was not retrievable

17. Robinson SN (1993) Orthodontic treatment and the wind instrument. Dent Update. 20:116-120.

► Excluded after full text reading, because of being a review paper

18. Mole C, Louis JP, Frey JM (1990) Physiological techniques of complete denture design for musicians, wind instrument players. Inf Dent. 72:2601-2606.

► Excluded based on title, because of addressing other topic

19. Ogino H (1990) The influence of playing the clarinet on the dentomaxillofacial morphology and function. Ou Daigaku Shigakushi 17:131-154.

► Excluded based on title and abstract, because of addressing the reverse question

20. Schönekerl H (1990) Experimental examinations about the influence of prosthetic therapeutic remedies for players on a wind-instrument as to the quality of the instrumental sound. Stomatol DDR. 40:88-90.

► Excluded based on title and abstract, because of addressing other topic

21. Rindisbacher T, Hirschi U, Ingervall B, Geering A (1990) Little influence on tooth position from playing a wind instrument. Angle Orthod. 60:223-228.

► Excluded based on title and abstract, because of addressing the reverse question

22. Brattström V, Odenrick L, Kvam E (1989) Dentofacial morphology in children playing musical wind instruments: a longitudinal study. Eur J Orthod. 11:179-185.

► Excluded based on title and abstract, because of addressing the reverse question

23. Grundig C (1982) Dental risk factors in woodwind and brass-wind instrument players. Stomatol DDR. 32:387-391.

► Excluded because full text was not retrievable

24. Herman E (1981) Influence of musical instruments on tooth positions. Am J Orthod. 80:145-155.

► Excluded based on title and abstract, because of addressing the reverse question

25. DiStasio ER (1981) Wind instruments: another look. J Mass Dent Soc. 30:152-155.

► Excluded based on title, because of being a review paper

26. De Roos P (1980) Some dental aspects of wind instrument playing. A preliminary report. Ned Tijdschr Tandheelkd. 87:299-302.

► Excluded after full text reading, because of addressing other topic

27. Gualtieri PA (1979) May Johnny or Janie play the clarinet? The Eastman Study: a report on the orthodontic evaluations of college-level and professional musicians who play brass and woodwind instruments. Am J Orthod. 76:260-276.

► Excluded based on title and abstract, because of addressing the reverse question

28. Lapter V, Kalousek M (1979). An orthodontoic approach to playing musical wind-instruments. Lijec Vjesn. 101:441-442.

► Excluded because full text was not retrievable

29. Ma HC, Laracuente JM (1979) The influence of playing musical wind instruments on oral tissues. Gen Dent. 27:46-50.

► Excluded based on title, because of addressing other topic

30. Shimada T (1978) A morphological study on the effect of wind instruments on the dento-oral region--with reference to the growing young people. J Nihon Univ Sch Dent. 20:23-36.

► Excluded based on title, because of addressing the reverse question

31. Haas W, Landeck E (1976) The question of stomatologic-neurological cooperation during diagnosis and therapy of functional disorders in the oral and perioral region in players of wind instruments. Stomatol DDR. 26:256-259.

► Excluded based on title, because of addressing other topic

32. Pang A (1976) Relation of musical wind instruments to malocclusion. J Am Dent Assoc. 92:565-570.

► Excluded based on title and abstract, because of addressing the reverse question

33. Landeck E, Riedrich P (1975) Teleradiodiagnosis for players of brass instruments. Stomatol DDR. 25:321-327.

► Excluded based on title and abstract, because of addressing other topic

34. Salzmann JA (1974) Editorial: Malocclusion, tongue thrusting, and wind instrument playing. Am J Orthod. 66:456-457.

► Excluded based on title, because of being an editorial

35. Herman E (1974) Orthodontic aspects of musical instrument selection. Am J Orthod. 65:519-530.

► Excluded after full text reading, because of being a review paper

36. Lovius BB, Huggins DG (1973) Orthodontics and the wind instrumentalist. J Dent. 2:65-68.

► Included

37. Landeck E, Weggen E (1973) Methodical auxiliary apparatus--an aid for the wind instrument player. Dtsch Stomatol. 23:438-443.

► Excluded based on title, because of addressing other topic

38. Kudrnác V, Zapletal L (1972) Changes of teeth of players of wind instruments. Cesk Stomatol. 72:177-182.

► Excluded based on title, because of addressing the reverse question

39. Schöttner HJ (1971) Studies on players of wind instruments. Dtsch Stomatol. 21:931-936.

► Excluded because full text was not retrievable

40. Ebersbach W (1971) Clinical-experimental studies on players of wind instruments about the effect of sagittal force on the periodontium of the incisors; therapeutic deductions. Dtsch Stomatol. 21:398-401.

► Excluded based on title, because of addressing other topic

41. Musil R, Oswald R (1969) Extension-mouth-pieaes for players of wood and brass wind instruments. Function and technical manufacturing instructions. Zahntechnik (Berl). 10:478-490.

► Excluded based on title, because of addressing other topic

42. Bielecka-Deluga L (1969) Types of mouthpieces, technics of playing on wind instruments and oral cavity environment. Protet Stomatol. 19:323-328.

► Excluded based on title, because of addressing other topic

43. Sand C (1969) Orthodontic, stomatologic and prosthetic problems for players of wind instruments. Acta Stomatol Belg. 66:143-206.

► Excluded because full text was not retrievable

44. Porter MM (1968) Dental problems in wind instrument playing. 11. Brass instruments. Br Dent J. 124:271-274.

► Excluded after full text reading, because of being a review paper

45. Porter MM (1968). Dental problems in wind instrument playing. 10. Brass instruments (continued) Br Dent J. 124:227-231.

► Excluded after full text reading, because of being a review paper

46. Porter MM (1968) Dental problems in wind instrument playing. 6--Single-reed instruments—The embouchure denture. Br Dent J. 124:34-36.

► Excluded based on title, because of addressing other topic

47. Thrun I, Romankiewicz GE (1966) Changes in the oral cavity caused by the playing of wind-instruments. Czas Stomatol. 19:831-837.

► Excluded based on title, because of addressing the reverse question

48. Engelman JA (1965) Measurement of perioral pressures during playing of musical wind instruments. Am J Orthod. 51:856-864.

► Excluded based on title, because of addressing other topic

49. Kessler HE (1965) Look for harmony: musical wind instruments and dental development. Dent Surv. 41:47-49.

► Excluded based on title, because of addressing the reverse question

50. Boehme W (1965) Current findings on the prosthetic management of musicians playing on wind instruments. Dtsch Stomatol. 15:179-183.

► Excluded based on title, because of addressing other topic

51. Schneider GA (1964) Dental measures and experiences in the treatment and rehabilitation of players of wind instruments. Z Gesamte Hyg. 10:260-271.

► Excluded based on title, because of addressing other topic

Additionally identified by Embase:

52. Fuhrimann S, Schüpbach A, Thüer U, Ingervall B (1987) Natural lip function in wind instrument players. European journal of orthodontics 9:216-223.

► Excluded based on title, because of addressing other topic

53. Farkas P (1986) Medical problems of wind players: A musician's perspective.

Cleveland Clinic Quarterly 53:33-37.

► Excluded based on title and abstract, because of being a review paper

54. Dunn RH (1982) Selecting a musical wind instrument for a student with orofacial muscle problems. International journal of orthodontics 20:19-22.

► Excluded based on title, because of addressing other topic

No studies were identified in the Cochrane Library
